# Two-factor higher-order model of perfectionism in Iranian general and clinical samples

**DOI:** 10.1186/s40359-021-00529-2

**Published:** 2021-02-17

**Authors:** Reza Moloodi, Abbas Pourshahbaz, Parvaneh Mohammadkhani, Ladan Fata, Ata Ghaderi

**Affiliations:** 1 Substance Abuse and Dependence Research Center, University of Social Welfare and Rehabilitation Sciences, Koodakyar St. Daneshjoo St., Tehran, Iran; 2 Department of Clinical Psychology, University of Social Welfare and Rehabilitation Sciences, Koodakyar St. Daneshjoo St., Tehran, Iran; 3 Medical Education and Development Center, Iran University of Medical Sciences, Hemmat Highway, Tehran, Iran; 4 Department of Clinical Neuroscience, Karolinska Institute, Stockholm, Sweden

**Keywords:** Perfectionism, Perfectionistic strivings, Perfectionistic concerns, Higher order construct, Iran

## Abstract

**Background:**

Studies in Western cultures have shown that perfectionism is conceptualized by two-factor higher-order model including perfectionistic strivings and perfectionistic concerns. However, little is known about the construct of perfectionism in Eastern societies. Thus, we examined the two-factor higher-order model of perfectionism in Iranian general and clinical samples.

**Methods:**

We recruited a general population sample (n = 384) and patients with major depressive disorder, obsessive compulsive disorder, social anxiety disorder, and eating disorders (n = 152) from Tehran, Iran from September 2016 to December 2017. They completed the Clinical Perfectionism Questionnaire, Perfectionism Inventory, and Depression, Anxiety, Stress Scale-21.

**Results:**

The two-factor higher-order model of perfectionism showed adequate fit with data for females from the general population and clinical sample. Data for males were only available from the general population, and the model showed adequate fit with the data first after removing the Rumination scale of the perfectionistic concerns. The perfectionistic strivings dimension showed no or negative association with depression, anxiety, and stress symptoms, but perfectionistic concerns dimension showed positive correlation with these indices in all samples for both males and females.

**Conclusions:**

The results support the two-factor higher-order model of perfectionism in samples of Iranian females from the general population and clinical sample. However, the results were different for males from the general population. In other words, the modified two-factor higher-order model showed acceptable fit with the data for males from the general population only after removing the Rumination scale from perfectionistic concerns. These differences among males and females were discussed.

## Background

A number of theoreticians discuss about conceptualization of perfectionism. Sigmund Freud [1] considered perfectionism as a unidimensional construct that was pathological in nature. Hamachek [2] was the first to introduce two types of perfectionism: normal and neurotic. He identified “normal perfectionists” as “those who derive a very real sense of pleasure from the labors of a painstaking effort and who feel free to be less precise as the situation permits” (p. 27). In contrast, he described neurotic perfectionists as “the people whose efforts never seem quite good enough. … they are unable to feel satisfaction because in their own eyes they never seem to do things good enough to warrant the feeling” (p. 27).

Following Hamachek [2], some researchers argued that perfectionism is multidimensional. Hewitt and Flett [3] identified Self-Oriented Perfectionism (SOP), Other Oriented Perfectionism (OOP), and Socially Prescribed Perfectionism (SPP). Also, Frost, Marten, Lahart, and Rosenblate [4] formulated perfectionism by a number of dimensions including Personal Standards (PS), Concern over Mistakes (CM), Doubt about Actions (DA), Parental Expectation (PE), Parental Criticism (PC), and Organization (Or). As an attempt to integrate views of Hewitt and Flett [3], and Frost et al. [4], Hill et al. [5] framed a model of perfectionism that captures Planfulness (Pl), Organization (Or), Striving for Excellence (SE), Concern over Mistakes (CM), Need for Approval (NA), High Standards for Others (HSO), Perceived Parental Pressure (PPP), and Rumination (Ru). These three research groups developed three separate measures to assess perfectionism based on their own model: Frost Multidimensional Perfectionism Scale (FMPS) [4], Hewit Multidimensional Perfectionism Scale (HMPS) [3], and Perfectionism Inventory [5].

The multidimensional conceptualization of perfectionism encountered two primary criticisms. First, factor analytic studies using these measures consistently showed that these dimensions loaded on two higher-order factors named “Perfectionistic Strivings” (PS), and “Perfectionistic Concerns” (PC) rather than multiple factors [6–8]. And these two higher order factors showed different patterns of relationship with indices of psychopathology and wellbeing. Perfectionistic concerns showed association with psychopathology indices such as depression, anxiety, and eating disorder symptoms (see Egan et al. for a review [9]), and poorer health [10]. Perfectionistic strivings correlated positively with experience of positive affect [11, 12], but showed weak or negative relationship with psychopathology [13, 14]. These distinct relationship patterns are in line with two-factor higher-order model of perfectionism. In addition, patients with Major Depressive Disorder (MDD) [15], Social Anxiety Disorder (SAD) [16], Obsessive–Compulsive Disorder (OCD) [17] and Eating Disorders (EDs) [18] scored higher on scales such as CM, DA, and SPP that constitute perfectionistic concerns than healthy control subjects. On the other hand, these studies did not find significant differences between clinical groups and healthy controls on scales such as SOP and PS that loaded on perfectionistic strivings. Second, some researchers suggested that various proposed dimensions (e.g. Parental Expectation, Parental Criticism, and Doubt about Actions) might be variables that simply correlate with perfectionism or outcome, but are not a genuine part of the perfectionism concept [13, 19, 20]. In addition, Stoeber and Damian [21] and Stoeber [22] discussed other oriented perfectionism is better considered as a form of perfectionism outside the two-factor model because it is directed at others.

Based on the second criticism, Shafran et al. [23] argued against multidimensional, and two-dimensional perspectives on perfectionism and introduced the concept of “clinical perfectionism” as a unidimensional construct. Clinical perfectionism referred to “the overdependence of self-evaluation on the determined pursuit of personally demanding, self-imposed standards in at least one highly salient domain, despite adverse consequences” [23] (p. 778). This definition was “strongly in favor of returning to a unidimensional approach to the study of perfectionism” [20] (p 1223) with heavy emphasis on the perfectionistic strivings. They devised a measure named Clinical Perfectionism Questionnaire (CPQ) to measure clinical perfectionism. However, exploratory and confirmatory factor analytic studies using the CPQ indicated that “clinical perfectionism” is better described by a two-dimensional approach: Personal Standards (PS) and Evaluative Concerns (EC) [21, 24, 25]. Finally, Egan et al. [26] reported that exploratory factor analysis revealed a two-factor solution for CPQ in both community and eating disorder samples.

Interestingly, most of the research concerning the construct of perfectionism come almost completely from Western cultures and little is known about the construct of perfectionism among Eastern societies. To our knowledge, a few studies compared the Eastern and Western samples in terms of perfectionism. Smith et al. [27] have made comparisons between Chinese and Canadian students with regard to the construct of perfectionism. They found that the two-factor higher-order model of perfectionism (i.e., perfectionistic strivings and perfectionistic concerns) fit with the data in both groups, and the path coefficients were invariant across Canadian and Chinese samples. A recent study compared Middle Eastern students with US students with regard to perfectionism [28]. The authors reported that Middle Eastern students scored significantly higher than US students on Parental Expectations and Self-Oriented Perfectionism. Given the mixed findings and some significant differences, it is not plausible to assume that findings on perfectionism construct in West always generalize to Eastern populations. Investigating the exact nature of a perfectionism within different cultural contexts has important theoretical, research-related, and clinical implications. It helps to understand and define the construct in a culturally sensitive manner, which in turn determine what should be measured in research and what must be targeted in clinical practice.

Thus, the aim of this cross-sectional study was to explore the higher order factor of perfectionism among Iranian females and males from the general population, and patients with psychological problems in clinical settings. Based on the evidence on two-factor higher-order model of perfectionism in Western cultures and findings of smith et al. [27], we predicted that perfectionism among Iranian general population and clinical sample consisted of two higher order factors: (1) Perfectionistic Strivings (PS) (consisted of Personal Standards from CPQ, as well as Organization, Striving for Excellence, and Planfulness from PI), and (2) Perfectionistic Concerns (PC) (consisted of Evaluative Concern from CPQ, as well as Concern over Mistakes, Need for Approval, and Rumination from PI). We omitted Perceived Parental Pressure (PPP) scale from the model based on the previous literature that announced it is not a genuine part of perfectionism [12, 13]. In addition, based on Stoeber [22] who showed other oriented perfectionism is better considered as a form of perfectionism outside the two-factor model, the High Standards for Others (HSO) scale was excluded from the model. We anticipated that the PC would positively correlate with symptoms of depression, anxiety, and stress, while PS would show no relationship or significant negative relationship with these symptoms.

## Methods

### Participants

The present study was part of a larger cross-sectional project investigating the etiological and maintaining mechanisms of perfectionism through structural equation modeling. The participants were recruited from the general population and clinics. The general population sample included 403 participants (204 females) in Tehran, Iran. They were selected via proportional quota sampling based on the last census data of Statistical Center of Iran (Statistical Centre of Iran, 2011). Proportional quota sampling is a type of non-random sampling. It sometimes referred to as a non-probability sampling method. Proportional quota sampling is usually utilized in surveys and opinion polls, where the total number of people to be surveyed is typically decided in advance. In this method, the sample was split between distinct subgroups or strata. Inclusion criteria were being between 18- and 50-years old, having completed high school, and living in Tehran for at least 6 previous years. Nineteen participants had skipped more than 10% of the items. Therefore, the data of 384 subjects (187 females) were analyzed.

The clinical sample consisted of 152 patients with MDD (n = 40, females = 26), OCD (n = 39, females = 24), SAD (n = 35, females = 26), or EDs (n = 38 females, bulimia nervosa = 31, anorexia nervosa = 7).

Monte Carlo Simulation Studies suggest that SEM models could be safely evaluated with small samples [29].They generally proposed a sample size of 150 to 200 people is sufficient for confirmatory factor analysis studies. Therefore, in the present study, we determined the sample size of *N* = 400 for the general population and *N* = 150 for the clinical sample.

### Material and procedure

The research procedure was approved by Ethical Review Board of University of Social Welfare and Rehabilitation sciences. All participants provided written consent. In order to gather data from general population sample, five social workers selected participants according to quota sampling matrix from visitors in health centers, parks, and/or cultural houses of Tehran, Iran. Participants were asked to complete a battery of questionnaires.

The clinical sample consisted of patients referred to the first author (R.M.) by psychiatrists or clinical psychologists for an evaluation using the Structured Clinical Interview for DSM-IV (SCID), who met the inclusion criteria and agreed to participate in the study. They were asked to complete a series of questionnaires and to return them to the researchers within one week.

### Clinical Perfectionism Questionnaire (CPQ)

The CPQ [30] assesses cognitive, emotional and behavioral components of clinical perfectionism over the past month by means of 12 items that are responded using a four-point Likert scale (from “not at all” to “all of the time”). A number of studies have demonstrated the validity and reliability of CPQ and indicated that the it captures two factors named Personal Standards (PS) and Evaluative Concern (EC) [25, 26]. The example of the items is as follow: “Have you been told that your standards are too high?” (PS factor); “Have you been afraid that you might not reach your standards?” (EC factor). As shown in the results section, Table 2, subscales of the CPQ showed acceptable McDonald's Omega coefficient. The CPQ can be seen in Additional file 1.

### Perfectionism Inventory (PI)

Hill et al. [5] developed a 59-item PI in order to combine and capture the core structures of FMPS and HMPS. Subjects are asked to respond on a 5-point scale from strongly disagree to strongly agree. The PI consisted of 8 subscales including Concern over Mistakes (CM) (e.g., “To me, a mistake equals failure”), Need for Approval (NA) (e.g., “I am over-sensitive to the comments of others”), Rumination (Ru) (e.g., “If I make a mistake, my whole day is ruined”), High Standards for Others (HSO) (e.g., “I’m often critical of others”), Perceived Parental Pressure (PPP) (e.g., “My parents are difficult to please”,) Organization (Or) (e.g., “I like to always be organized and disciplined”), Planfulness (Pl) (e.g., “I find myself planning many of my decisions”), and Striving for Excellence (SE) (e.g., “I have to be the best in every assignment I do”). The exploratory principal components analysis resulted in a two higher order factor solution called “Conscientious Perfectionism” (based on Or, SE, Pl, and HSO) and “Evaluative Perfectionism” (based on CM, Ru, NA, and PPC). Jamshidi et al. [31] reported satisfactory structural validity, convergent validity, and internal consistency of the Persian version of PI. As illustrated in the results section, Table 2, all subscales of the PI showed acceptable to good McDonald's Omega coefficient. The PI can be seen in Additional file 1.

### Depression Anxiety Stress Scales-21 (DASS-21)

The DASS-21 is a self-report instrument consisted of three subscales that measure symptoms of depression (e.g., “I felt down-hearted and blue”), anxiety (e.g., “I felt I was close to panic”), and stress (e.g., “I found myself getting agitated”) over the past week. Participants were asked to answer the items using a 0 (did not apply to me at all) to 3 (apply to me very much) scale [32]. The Persian version of the DASS-21 has acceptable construct and convergent validity as well as internal consistency [33]. Internal consistency of DASS-21 and in its subscales in general population were as follow: DASS-21 total = 0.92; Depression = 0.85; Anxiety = 0.81; Stress = 0.83. Among clinical sample, internal consistency was as follow: DASS-21 total = 0.92; Depression = 0.84; Anxiety = 0.83; Stress = 0.87. The DASS-21 can be seen in Additional file 1. 

### Statistical analysis

Confirmatory Factor Analysis (CFA) with maximum likelihood estimation and fixing a factor loading method was performed using AMOS 23 [34] to test the two-factor higher-order model of perfectionism. To establish the fit of the model, we considered the χ^2^/df-ratio less than 3, as well as Goodness-of-Fit Index (GFI), Adjusted Goodness-of-Fit Index (AGFI), Incremental Fit Index (IFI), Comparative Fit Index (CFI) with cut off ≥ 0.95 as acceptable [35]. We Also considered the Root Mean Square Error of Approximation (RMSEA), and Standardized Root Mean Square Residual (SRMR) with values ≤ 0.08 indicating adequate fit [35]. Internal consistency of subscales of CPQ and PI were assessed using McDonald's Omega coefficient. The association of PS and PC Higher order factors with indices of depression, anxiety, and stress were assessed by Pearson correlation coefficient.

## Results

### Demographic information

In general population sample, the mean age of the males was 33.23 (SD = 9.18) and females 32.71 (SD = 9.78). The mean age of the four clinical groups were as follow: MDD = 29.87 (*SD* = 5.94); OCD = 31.25 (*SD* = 5.52); SAD = 28.37 (*SD* = 6.37); and EDs = 30.38 (*SD* = 5.55). Further demographic characteristics of general population and clinical sample are illustrated in Table 1.

**Table 1:** Demographic information for the sample from the general population and the clinical sample

	General population (n = 384)		Clinical sample (n = 152)			
	Men (n = 197)	Women (187)	MDD (n = 40)	OCD (n = 39)	SAD (n = 35)	EDs (n = 38)
	*N* (%)	*N* (%)	*N* (%)	*N* (%)	*N* (%)	*N* (%)
Education						
Completed high school	94 (47.71%)	94 (50.26%)	14 (35%)	14 (35.9%)	12 (34.28%)	8 (21.05%)
Completed college	24 (12.18%)	33 (17.64%)	1 (2.5%)	0 (0%)	0 (0%)	1 (2.63%)
Bachelor	60 (30.45%)	51 (27.27%)	12(30%)	12 (31%)	10 (28.57%)	11 (28.94%)
Master degree	16 (8.12%)	7 (3.74%)	10 (25%)	9 (23.1%)	9(25.71%)	10 (26.31%)
PhD	3 (1.52%)	2 (1.06%)	3 (7.5%)	4 (10.3%)	4 (11.42%)	8 (21.05%)
Occupation						
Student	44 (22.3%)^a^	52 (27.80%)	5 (12.5%)	12 (31%)	15 (42.85%)	11 (28.94%)
Housewife	0 (0%)	45 (24.06%)	4 (10%)	2 (5.1%)	2 (5.7%)	0 (0%)
Self-employed	62 (31.5%)	23 (12.29%)	11 (27.5%)	10 (25.56%)	7 (20%)	9 (23.68%)
Employee	55 (27.9%)	63 (33.7%)	17 (42.5%)	13 (33.5%)	11 (31.42%)	17 (44.73%)
Unemployed	15 (7.6%)	2 (1.06%)	1 (2.5%)	2 (5.1%)	0 (0%)	1 (2.63%)
Retired	0 (0%)	2 (1.06%)	0 (0%)	0 (0%)	0 (0%)	0 (0%)

^a^21 men did not report their occupation status

### Measurement model

In order to test the dimensionality of each subscale, independent CFA was performed to examine the measurement model of Concern over Mistakes, Need for Approval, Evaluative Concern, Rumination, Planfulness, Organization, Personal Standards, and Striving for Excellence subscales. All factor loadings of the models were significant and all subscales showed unidimensional construct. The McDonald's Omega of the subscales ranged from 0.75 to 0.86 showing satisfactory internal consistency (Table 2).

**Table 2:** Measurement model and McDonald's Omega of subscales

Subscales	General population				Clinical sample	
	Males		Females			
	Fit indices	Omega	Fit indices	Omega	Fit indices	Omega
Concern over mistakes	χ^2^(20, N = 197) = 30.16, *p* > *.*05	.78	χ^2^(20, N = 187) = 38.6, *p* < *.*05	.76	χ^2^(20, N = 152) = 76.78, *p* < *.*0001	.78
	χ^2^/df-ratio = 1.50		χ^2^/df-ratio = 1.93		χ^2^/df-ratio = 3.83	
	*GFI* = .96		*GFI* = .95		*GFI* = .95	
	*AGFI* = .93		*AGFI* = .91		*AGFI* = .90	
	*IFI* = .97		*IFI* = .97		*IFI* = .97	
	*CFI* = .97		*CFI* = .97		*CFI* = .97	
	*RMSEA* = .05		*RMSEA* = .07		*RMSEA* = .07	
	SRMR = 0.03		SRMR = 0.05		SRMR = 0.05	
Need for approval	χ^2^(18, N = 197) = 27.61, *p* > *.*05	.81	χ^2^(18, N = 187) = 29.43, *p* < *.*05	.80	χ^2^(18, N = 152) = 22.18, *p* > *.*05	.80
	χ^2^/df-ratio = 1.53		χ^2^/df-ratio = 1.63		χ^2^/df-ratio = 1.23	
	*GFI* = .96		*GFI* = .96		*GFI* = .96	
	*AGFI* = .93		*AGFI* = .92		*AGFI* = .93	
	*IFI* = .98		*IFI* = .98		*IFI* = .99	
	*CFI* = .98		*CFI* = .98		*CFI* = .99	
	*RMSEA* = .05		*RMSEA* = .05		*RMSEA* = .03	
	SRMR = 0.05		SRMR = 0.05		SRMR = 0.03	
Evaluative concern	χ^2^(2, N = 197) = 1.21, *p* > *.*05	.76	χ^2^(2, N = 187) = 0.17, *p* > *.*05	.77	χ^2^(2, N = 187) = 1.05, *p* > *.*05	.79
	χ^2^/df-ratio = 0.60		χ^2^/df-ratio = 0.08		χ^2^/df-ratio = 0.52	
	*GFI* = .99		*GFI* = 1.00		*GFI* = .99	
	*AGFI* = .98		*AGFI* = .99			
	*IFI* = .99		*IFI* = 1.00		*IFI* = .98	
	*CFI* = .99		*CFI* = 1.00		*CFI* = .98	
	*RMSEA* = .000		*RMSEA* = .000		*RMSEA* = .000	
	SRMR = 0.01		SRMR = 0.01		SRMR = 0.01	
Rumination	χ^2^(12, N = 197) = 18.35, *p* > *.*05	.79	χ^2^(12, N = 187) = 21.96, *p* < *.*05	.78	χ^2^(12, N = 152) = 18.12, *p* > *.*05	.79
	χ^2^/df-ratio = 1.52		χ^2^/df-ratio = 1.83		χ^2^/df-ratio = 1.51	
	*GFI* = .97		*GFI* = .96		*GFI* = .95	
	*AGFI* = .94		*AGFI* = .92		*AGFI* = .91	
	*IFI* = .98		*IFI* = .97		*IFI* = .98	
	*CFI* = .98		*CFI* = .97		*CFI* = .98	
	*RMSEA* = .05		*RMSEA* = .06		*RMSEA* = .05	
	SRMR = 0.04		SRMR = 0.04		SRMR = 0.04	
Planfulness	χ^2^(12, N = 197) = 18.88, *p* > .05	.81	χ^2^(12, N = 187) = 24.33, *p* < .01	.83	χ^2^(12, N = 152) = 24.33, p < .01	.80
	χ^2^/df-ratio = 1.57		χ^2^/df-ratio = 2.02		χ^2^/df-ratio = 2.02	
	*GFI* = .97		*GFI* = .96		*GFI* = .94	
	*AGFI* = .93		*AGFI* = .91		*AGFI* = .91	
	*IFI* = .98		*IFI* = .96		*IFI* = .95	
	*CFI* = .98		*CFI* = .96		*CFI* = .95	
	*RMSEA* = .05		*RMSEA* = .07		*RMSEA* = .08	
	SRMR = 0.04		SRMR = 0.04		SRMR = 0.06	
Organization	χ^2^(19, N = 197) = 39.59, *p* < *.*001	.86	χ^2^(19, N = 187) = 37.62, *p* < *.*001	.86	χ^2^(19, N = 152) = 23*.*74, *p* > .05	.86
	χ^2^/df-ratio = 2.08		χ^2^/df-ratio = 1.98		χ^2^/df-ratio = 1.25	
	*GFI* = .94		*GFI* = .95		*GFI* = .96	
	*AGFI* = .90		*AGFI* = .90		*AGFI* = .93	
	*IFI* = .97		*IFI* = .97		*IFI* = .99	
	*CFI* = .97		*CFI* = .97		*CFI* = .99	
	*RMSEA* = .07		*RMSEA* = .07		*RMSEA* = .04	
	SRMR = 0.05		SRMR = 0.03		SRMR = 0.02	
Personal standards	χ^2^(14, N = 197) = 37.74, *p* > .05	.78	χ^2^(14, N = 187) = 11.18, *p* > .05	.73	χ^2^(14, N = 187) = 13.16, *p* > .05	.75
	χ^2^/df-ratio = 2.69		χ^2^/df-ratio = 0.79		χ^2^/df-ratio = 0.94	
	*GFI* = .98		*GFI* = .98		*GFI* = .97	
	*AGFI* = .95		*AGFI* = .96		*AGFI* = .93	
	*IFI* = .98		*IFI* = 1.00		*IFI* = .96	
	*CFI* = .98		*CFI* = 1.00		*CFI* = .96	
	*RMSEA* = .03		*RMSEA* = .000		*RMSEA* = .04	
	SRMR = 0.04		SRMR = 0.02		SRMR = 0.03	
Striving for excellence	χ^2^(9, N = 197) = 20.20, *p* < .05	.78	χ^2^(9, N = 187) = 12.61, *p* > .05	.76	χ^2^(9, N = 197) = 13.05, *p* > .05	.78
	χ^2^/df-ratio = 2.24		χ^2^/df-ratio = 1.40		χ^2^/df-ratio = 1.45	
	*GFI* = .96		*GFI* = .97		*GFI* = .97	
	*AGFI* = .92		*AGFI* = .95		*AGFI* = .92	
	*IFI* = .96		*IFI* = .98		*IFI* = .98	
	*CFI* = .96		*CFI* = .98		*CFI* = .98	
	*RMSEA* = .08		*RMSEA* = .04		*RMSEA* = .05	
	SRMR = 0.04		SRMR = 0.03		SRMR = 0.03	

### Confirmatory factor analysis of two-factor higher-order model of perfectionism

**Fig. 1:**
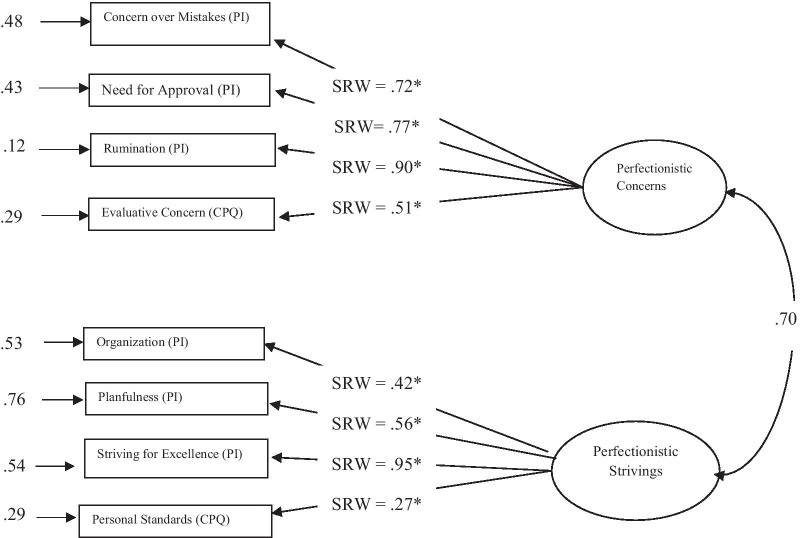
Higher order two dimensional model of perfectionism in the clinical sample. *CPQ* Clinical Perfectionism Questionnaire, *PI* perfectionism inventory, *SRW *standardized regression weight, *p < 0.01

Analysis of clinical data showed that two-factor higher-order model of perfectionism with a from perfectionistic strivings higher order factor (including Planfulness, Organization, Personal Standards, and Striving for Excellence subscales) and perfectionistic concerns higher order factor (including Concern over Mistakes, Need for Approval, Evaluative Concern, and Rumination subscales) showed adequate fit (*χ*^2^(19, N = 152) = 34.01, *p* = 0.02, χ^2^/df-ratio = 1.79, *GFI* = 0.95, *AGFI* = 0.91, *IFI* = 0.97, *CFI* = 0.97, *RMSEA* = 0.06, 90% CI [0.02, 0.10]), and SRMR = 0.05) (Fig. 1).

In the sample from the general population the two-factor higher-order model resulted in poor fit (*χ*^2^(19, N = 384) = 246.62, *p* < 0.0001, χ^2^/df-ratio = 12.98, *GFI* = 0.90, *AGFI* = 0.75, *IFI* = 0.88, *CFI* = 0.88, *RMSEA* = 0.14, 90% CI [0.12, 0.16], and SRMR = 0.11). Scrutinizing data indicated that the factor loading of the Rumination scale on perfectionistic concerns was not significant (t = 1.89, *p* = 0.08). Conducting CFA without Rumination scale resulted in relatively adequate fit (*χ*^2^(13, N = 384) = 67.86, *p* < 0.0001, χ^2^/df-ratio = 5.22, *GFI* = 0.96, *AGFI* = 0.90, *IFI* = 0.94, *CFI* = 0.94, *RMSEA* = 0.09, 90% CI [0.07, 0.12]). Given the evidence for the gender differences on rumination [36], we decided to run the CFA separately for males and females.

**Fig. 2:**
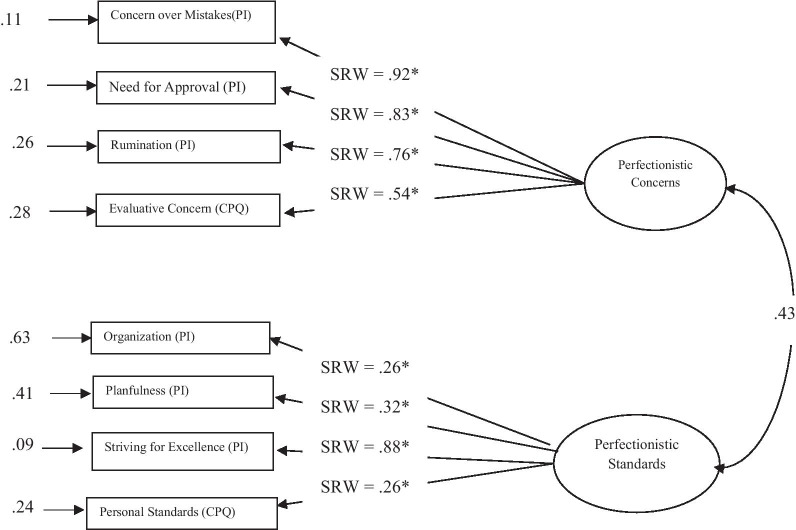
Higher order two dimensional model of perfectionism among women from general population. *CPQ* Clinical Perfectionism Questionnaire, *PI* perfectionism inventory, *SRW* standardized regression weight, *p < 0.01

**Fig. 3:**
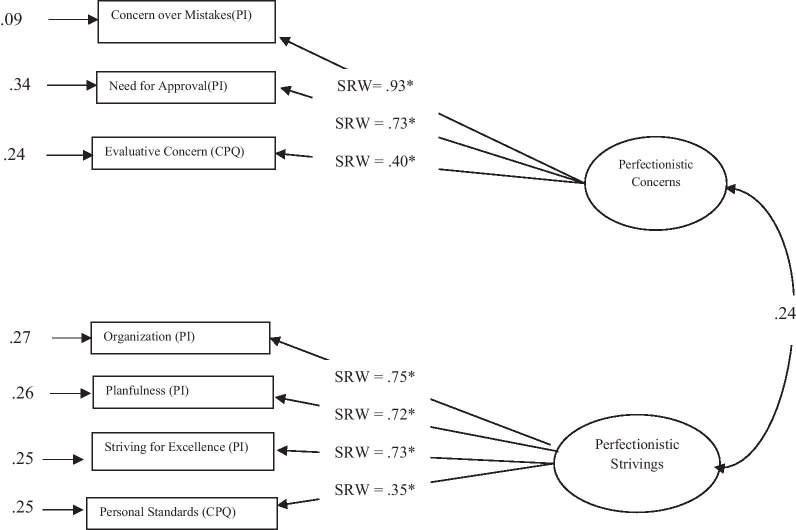
Modified higher order two dimensional model of perfectionism among general population men. *CPQ* Clinical Perfectionism Questionnaire, *PI *perfectionism inventory, *SRW *standardized regression weight, *p < 0.01

The two-factor higher-order model for females from the general population showed good fit (*χ*^2^(19, N = 187) = 50.92, *p* < 0.0001, χ^2^/df-ratio = 2.68, *GFI* = 0.94, *AGFI* = 0.88, *IFI* = 0.94, *CFI* = 0.94, *RMSEA* = 0.09, 90% CI [0.05, 0.12], SRMR = 0.06) (Fig. 2). On the other hand, CFA of a corresponding model for males indicated lack of fit (*χ*^2^(19, N = 197) = 115*.*9, *p* < 0.001, χ^2^/df-ratio = 6.10, *GFI* = 0.89, *AGFI* = 0.77, *IFI* = 0.85, *CFI* = 0.85, *RMSEA* = 0.16, 90% CI [0.13, 0.19], and SRMR = 0.11). Rumination scale was the source of problem as its factor loading on Perfectionistic Concerns was not significant (*t* = 1.20, *p* = 0.22). After removing the Rumination scale, model for males showed good fit with data (*χ*^2^(13, N = 197) = 37.05, *p* < 0.001, χ^2^/df-ratio = 2.85, *GFI* = 0.95, *AGFI* = 0.89, *IFI* = 0.94, *CFI* = 0.94, *RMSEA* = 0.09, 90% CI [0.06, 0.13]), and SRMR = 0.09 (Fig. 3).

### Relationship of Perfectionistic Strivings and Perfectionistic Concerns with depression, anxiety, and stress symptoms

In order to test the relationship pattern of Perfectionistic Strivings and Perfectionistic Concerns with depression, anxiety, and stress the Pearson correlation coefficient was used (Table 3). Among females from general population and clinical samples, Perfectionistic Strivings was not significantly correlated with depression, anxiety, stress and DASS-21 total scores. However, among males from general population, Perfectionistic Strivings showed negative significant correlation with indices of depression, anxiety, stress, and DASS total scores. In all three groups, Perfectionistic Concerns was significantly associated with depression, anxiety, stress, and DASS-21 total scores.

**Table 3:** Correlation of personal standards and evaluative concerns with depression, anxiety, stress and DASS-21 total scores

	Women from general population (n = 187)		Men from General population (n = 198)		Clinical sample (n = 152)	
	PS	PC	PS	PC	PS	PC
Depression	− .08	.55*	− .32*	.34*	.09	.48*
Anxiety	− .01	.40*	− .23	.30*	.03	.32*
Stress	.03	.55*	− .26*	.35*	.12	.47*
DASS-21 total	− .02	.56*	− .30*	.37*	.08	.48*

Perfectionistic Strivings is the average of Planfulness, Organization, Personal Standards, and Striving for Excellence subscale scores. Perfectionistic Concerns is the average of Concern over Mistakes, Need for Approval, Evaluative Concern, and Rumination subscales (Rumination subscale was excluded for men from general population)

*PS* perfectionistic strivings, *PC* perfectionistic concerns

*p < .001

## Discussion

Testing—rather that assuming- the generalizability of the psychological models that have been developed in Western cultures is a clinical and research necessity. Researchers and clinicians would have access to standardized and culturally sensitive measures derived from those models, which contributes to more reliable and valid research and clinical practice. If researchers and practitioners simply assume the cross-cultural generalizability of the models, they may ignore the unique characteristics of the cultural contexts [27]. Thus, the primary aim of the present study was to explore the higher order dimensions of perfectionism in Iranian general population and clinical samples. As far as we know, this was the third study that investigate the construct of perfectionism concept in an Eastern society after smith et al. [6] and Walton et al. [28].

The results of CFA for females from the general population and clinical sample showed that two-factor higher-order model (consisting of perfectionistic strivings and perfectionistic concerns) fit with data. These results are consistent with previous researches that support a two-factor higher-order model of perfectionism in various Western populations [4, 6–8]. These findings implied that the two-factor higher-order model of perfectionism was generalizable to Iranian females from both the general population and clinical samples. However, the results were slightly different for males from the general population. The CFA showed that the modified two-factor higher-order model showed acceptable fit with the data for males only after removing the Rumination scale from perfectionistic concerns. In other words, among Iranian males from the general population rumination does not make a significant contribution to perfectionistic concerns. Jamshidi and colleagues [31] found that among Iranian high school students (149 females and 164 males) Rumination did not load on perfectionistic concerns in their factor analysis of higher order structure of Perfectionism Inventory. Unfortunately, they did not report the results of male and female students separately. Consistent with our findings, a more recent study of Iranian female college students (n = 832) indicated that Rumination has most robust factor loading on perfectionistic concerns [37]. Although a gender difference in rumination has been shown in previous researches in other contexts [36, 38], the findings in our study might also to some extent reflect a paternal culture within which rumination is considered a female habit, and males are encouraged not to think too much about past events, future uncertainties, or failures. Unfortunately, we are not aware of any previous study of the higher order construct of perfectionism among other Eastern or Middle-East populations. The construct of perfectionistic concerns might in essence be different among Iranian men. While perfectionistic males and females are similarly concerned about their future failures or mistakes (e.g., Concern over Mistakes, and Evaluative Concern subscales), and they are very sensitive to others’ opinion on their achievements or failures (e.g., Need for Approval subscale), it seems that males are less prone to think about mistakes than females (e.g., Item 24 of the PI “If I make a mistake, my whole day is ruined”). Thus, future researches should further explore gender differences in construct of perfectionistic concerns in Eastern societies. One question that remains unanswered is whether rumination is a central component of perfectionism or simply a correlate. This question was already raised in relation to other dimensions of perfectionism (e.g. Parental Expectation, Parental Criticism, and Doubt about Actions) [13, 19, 23]. Therefore, future research should investigate the precise role of rumination with regard to perfectionism and its dimensions.

The pattern of relationships of Perfectionistic Concerns and Perfectionistic Strivings dimensions with symptoms of depression, anxiety, and stress verified the two-factor higher-order model of perfectionism in Iran. Among females from the general population and clinical sample, the Perfectionistic Strivings showed no relationship with psychopathological indices. While, among males from the general population, the Perfectionistic Strivings correlated with lower level of depression, anxiety, and stress. This pattern of correlations is highly consistent with findings of previous studies on Western cultures [9, 14, 39]. In addition, the findings might suggest the cross-cultural consistency of the perfectionism in Western and Iranian populations. On the other hand, the different relationship pattern of Perfectionistic Strivings with psychopathological indices among males and females might be due to the differences in social expectations on Iranian males and females. Traditionally, Iranian culture expects males to have high goals and standards on education, work, and money-making so they could take on full financial responsibility for their family. While increasing opportunities to professional and educational achievement for females are provided nowadays, some cultural norms still expect females to concentrate on traditional female roles (i.e., raising children and taking care of the household). Males are to a higher extent encouraged to achieve high perfectionistic standards. Another potential explanation might be that some self-report instruments of perfectionism are based on the societal norms for middle class white males, while other instruments such as those capturing body image are typically developed based on norms for middle class white females. Consequently, the former instruments might fail to capture the contextually specific targets of perfectionism that might be more specific to traditionally female than male roles, and thus produce gender differences that are an artifact of measurement. Perfectionistic Concerns significantly correlated with depression, anxiety and stress among both general population and clinical sample.

A notable strength of the study is the size of the samples from the general and clinical populations. However, the results should be interpreted with regard to its limitations. First, the study carried out among those between 20 and 50 years old and could not be generalizable to Iranian younger and older people. Second, in light of the lack of valid information about reliability and validity of Persian version of HMPS and FMPS, we could not use these measures in the present study. Lack for such instruments hindered us to assess the construct of perfectionism in a comprehensive manner in Iranian population. In addition, our results cannot be adequately compared with previous literature that used HMPS and FMPS. Therefore, future researches should concentrate on investigating psychometric properties of HMPS and FMPS and then replicate this study with those instruments. Third, the absence of measures of wellbeing or life satisfaction was another limitation of the study. Examining relationship of perfectionistic strivings and perfectionistic concerns with wellbeing, quality of life, or social adjustment indices would clarify more functional or dysfunctional nature of each dimension of perfectionism. Thus, future researches should investigate the relationship pattern of perfectionistic strivings and perfectionistic concerns with wellbeing, quality of life, or social adjustment indices. Lastly, we could not compare the higher-order model of perfectionism among males and females of clinical sample because of small sample size of males in clinical sample. Gender differences might be highly present and culturally different from one culture to another. Therefore, future studies should investigate potential gender differences in clinical groups.

## Conclusion

The main conclusion of this research is that perfectionism could be conceptualized by two-factor higher-order model including perfectionistic strivings and perfectionistic concerns among Iranian women of general population and clinical samples. However, the results indicated that among Iranian men of general population, Rumination might not be a genuine component of the perfectionistic concerns. Therefore, future research should explore the precise role of Rumination in the perfectionism. The results of the present study indirectly implied that parents and teachers should pay attention to the differences of positive and negative perfectionism and try their best to form positive perfectionism in the children. Also, current findings can help Iranian researchers and psychotherapists to assess and conceptualize negative and positive perfectionism astutely.

## Supplementary Information

**Additional file 1.** Clinical Perfectionism Questionnaire, Perfectionism Inventory, and Depression, Anxiety, Stress Scale-21.

## Data Availability

University of Social Welfare and Rehabilitation Sciences has approved and supported that only researchers of the manuscript will have access to the dataset, so the data used in this study is not available for public view. Still, requests can be written officially to the university.

## Abbreviations

SOP: Self-oriented perfectionism
OOP: Other oriented perfectionism
SPP: Socially prescribed perfectionism
PS: Personal standards
CM: Concern over mistakes
DA: Doubt about actions
PE: Parental expectation
PC: Parental criticism
Or: Organization
SE: Striving for excellence
NA: Need for approval
HSO: High standards for others
PPP: Perceived parental pressure
Ru: Rumination
FMPS: Frost Multidimensional Perfectionism Scale
HMPS: Hewit Multidimensional Perfectionism Scale
PS: Perfectionistic strivings
PC: Perfectionistic concerns
MDD: Major depressive disorder
SAD: Social anxiety disorder
OCD: Obsessive–compulsive disorder
EDs: Eating disorders
CPQ: Clinical Perfectionism Questionnaire
PS: Personal standards
EC: Evaluative concerns
DASS-21: Depression Anxiety Stress Scales-21
CFA: Confirmatory factor analysis
GFI: Goodness-of-Fit Index
AGFI: Adjusted Goodness-of-Fit Index
IFI: Incremental Fit Index
CFI: Comparative Fit Index
RMSEA: Root mean square error of approximation
SRMR: Standardized root mean square residual
