# Supplementary material for: Two-factor higher-order model of perfectionism in Iranian general and clinical samples
Source: BMC Psychol. 2021 Feb 17;9:30. doi: 10.1186/s40359-021-00529-2 (PMC7890826; doi:10.1186/s40359-021-00529-2)
Supplement: Supplementary file 1 — Additional file 1. Clinical Perfectionism Questionnaire, Perfectionism Inventory, and Depression, Anxiety, Stress Scale-21. [file 40359_2021_529_MOESM1_ESM.docx]

**DASS21** Name: Date:

Please read each statement and circle a number 0, 1, 2 or 3 which indicates how much the statement applied to you **over the past week**. There are no right or wrong answers. Do not spend too much time on any statement.

The rating scale is as follows:

1. Did not apply to me at all
2. Applied to me to some degree, or some of the time
3. Applied to me to a considerable degree or a good part of time
4. Applied to me very much or most of the time

| 1 (s) | I found it hard to wind down | 0 | 1 | 2 | 3 |
| --- | --- | --- | --- | --- | --- |
| 2 (a) | I was aware of dryness of my mouth | 0 | 1 | 2 | 3 |
| 3 (d) | I couldn’t seem to experience any positive feeling at all | 0 | 1 | 2 | 3 |
| 4 (a) | I experienced breathing difficulty (e.g. excessively rapid breathing, breathlessness in the absence of physical exertion) | 0 | 1 | 2 | 3 |
| 5 (d) | I found it difficult to work up the initiative to do things | 0 | 1 | 2 | 3 |
| 6 (s) | I tended to over-react to situations | 0 | 1 | 2 | 3 |
| 7 (a) | I experienced trembling (e.g. in the hands) | 0 | 1 | 2 | 3 |
| 8 (s) | I felt that I was using a lot of nervous energy | 0 | 1 | 2 | 3 |
| 9 (a) | I was worried about situations in which I might panic and make a fool of myself | 0 | 1 | 2 | 3 |
| 10 (d) | I felt that I had nothing to look forward to | 0 | 1 | 2 | 3 |
| 11 (s) | I found myself getting agitated | 0 | 1 | 2 | 3 |
| 12 (s) | I found it difficult to relax | 0 | 1 | 2 | 3 |
| 13 (d) | I felt down-hearted and blue | 0 | 1 | 2 | 3 |
| 14 (s) | I was intolerant of anything that kept me from getting on with what I was doing | 0 | 1 | 2 | 3 |
| 15 (a) | I felt I was close to panic | 0 | 1 | 2 | 3 |
| 16 (d) | I was unable to become enthusiastic about anything | 0 | 1 | 2 | 3 |
| 17 (d) | I felt I wasn’t worth much as a person | 0 | 1 | 2 | 3 |
| 18 (s) | I felt that I was rather touchy | 0 | 1 | 2 | 3 |
| 19 (a) | I was aware of the action of my heart in the absence of physical exertion (e.g. sense of heart rate increase, heart missing a beat) | 0 | 1 | 2 | 3 |
| 20 (a) | I felt scared without any good reason | 0 | 1 | 2 | 3 |
| 21 (d) | I felt that life was meaningless | 0 | 1 | 2 | 3 |

**Perfectionism Inventory**

**Please use the following options to rate how much you generally agree with each statement.**

**1 2 3 4 5**

**strongly disagree disagree somewhat neither agree agree somewhat strongly agree**

**nor disagree**

1. My work needs to be perfect, in order for me to be satisfied. (se1)
2. I am over-sensitive to the comments of others. (na1)
3. I usually let people know when their work isn’t up to my standards. (hso1)
4. I am well-organized. (o1)
5. I think through my options carefully before making a decision. (p1)
6. If I make mistakes, people might think less of me. (cm1)
7. I’ve always felt pressure from my parent(s) to be the best. (pp1)
8. If I do something less than perfectly, I have a hard time getting over it. (r1)
9. All my energy is put into achieving a flawless result. (se2)
10. I compare my work to others and often feel inadequate. (na2)
11. I get upset when other people don’t maintain the same standards I do. 2)
12. I think things should be put away in their place. (o2)
13. I find myself planning many of my decisions. (p2)
14. I am particularly embarrassed by failure. (cm2)
15. My parents hold me to high standards. (pp2)
16. I spend a lot of time worrying about things I’ve done, or things I need to do. (r2)
17. I can’t stand to do something halfway. (se3)
18. I am sensitive to how others respond to my work. (na3)
19. I’m not very patient with people’s excuses for poor work. (hso3)
20. I would characterize myself as an orderly person. (o3)
21. Most of my decisions are made after I have had time to think about them. (p3)
22. I over-react to making mistakes. (cm3)
23. My parent(s) are difficult to please. (pp3)
24. If I make a mistake, my whole day is ruined. (r3)
25. I have to be the best in every assignment I do. (se4)
26. I’m concerned with whether or not other people approve of my actions. (na4)
27. I’m often critical of others. (hso4)
28. I like to always be organized and disciplined. (o4)
29. I usually need to think things through before I know what I want. (p4)
30. If someone points out a mistake I’ve made, I feel like I’ve lost that person’s respect in some way. (cm4)
31. My parent(s) have high expectations for achievement. (pp4)
32. If I say or do something dumb I tend to think about it for the rest of the day. (r4)
33. I drive myself rigorously to achieve high standards. (se5)
34. I often don’t say anything, because I’m scared I might say the wrong thing. (na5)
35. I am frequently aggravated by the lazy or sloppy work of others. (hso5)
36. I clean my home often. (o5)
37. I need time to think up a plan before I take action. (p5)
38. If I mess up on one thing, people might start questioning everything I do. (cm5)
39. Growing up, I felt a lot of pressure to do everything right. (pp5)
40. When I make an error, I generally can’t stop thinking about it. (r5)
41. I must achieve excellence in everything I do. (se6)
42. I am self-conscious about what others think of me. (na6)
43. I have little tolerance for other people’s careless mistakes. (hso6)
44. I make sure to put things away as soon as I’m done using them. (o6)
45. I tend to deliberate before making up my mind. (p6)
46. To me, a mistake equals failure. (cm6)
47. My parent(s) put a lot of pressure on me to succeed. (pp6)
48. I often obsess over some of the things I have done. (r6)
49. I am often concerned that people will take what I say the wrong way. (na7)
50. I often get frustrated over other people’s mistakes. (hso7)
51. My closet is neat and organized. (o7)
52. I usually don’t make decisions on the spot. (p7)
53. Making mistakes is a sign of stupidity. (cm7)
54. I always felt that my parent(s) wanted me to be perfect. (pp7)
55. After I turn a project in, I can’t stop thinking of how it could have been better. (r7)
56. My workspace is generally organized. (o8)
57. If I make a serious mistake, I feel like I’m less of a person. (cm8)
58. My parent(s) have expected nothing but my best. (pp8)
59. I spend a great deal of time worrying about other people’s opinion of me.(na8)

Under no circumstances should this questionnaire be copied, distributed or quoted from without prior written permission of C. G. Fairburn.

| INSTRUCTIONS  This questionnaire is concerned with “perfectionism”. **By perfectionism, we mean trying to meet really high standards whether or not you actually succeed in reaching them.**  **In this questionnaire we are only concerned with perfectionism that affects areas of**  **life other than your eating, weight, or appearance.** | | | | | |
| --- | --- | --- | --- | --- | --- |
| Have you been trying to achieve high standards over the past month whether or not you have succeeded (excluding standards for your eating, weight or appearance)?  Please circle YES or NO.  **YES / NO** | | | | | |
| If so, in what areas of your life (other than eating, weight or appearance) has this applied?  - for example, it might have been in your performance at work, at sport, at music, at home, etc. Please note these below:  …………………………………………………………………………………………………  ………………………………………………………………………………………………… | | | | | |
| **Now, please place a ‘X’ in the column below which best describes you over the past month.**  Remember, do not count standards for your eating, weight or appearance. | | | | | |
|  | **Over the past month…….** | Not at all | Some of the time | Most of the time | All of the time |
| 1 | Over the past month, have you pushed yourself really hard to meet your goals? |  |  |  |  |
| 2 | Over the past month, have you tended to focus on what you have achieved, rather than on what you have not achieved? |  |  |  |  |
| 3 | Over the past month, have you been told that your standards are too high? |  |  |  |  |
| 4 | Over the past month, have you felt a failure as a person because you have not succeeded in meeting your goals? |  |  |  |  |
| 5 | Over the past month, have you been afraid that you might not reach your standards? |  |  |  |  |
| 6 | Over the past month, have you raised your standards because you thought they were too easy? |  |  |  |  |

Under no circumstances should this questionnaire be copied, distributed or quoted from without prior written permission of C. G. Fairburn.

|  | **Over the past month …….** | Not at all | Some of the time | Most of the time | All of the time |
| --- | --- | --- | --- | --- | --- |
| 7 | Over the past month, have you judged yourself on the basis of your ability to achieve high standards? |  |  |  |  |
| 8 | Over the past month, have you done just enough to get by? |  |  |  |  |
| 9 | Over the past month, have you repeatedly checked how well you are doing at meeting your standards (for example, by comparing your performance with that of others)? |  |  |  |  |
| 10 | Over the past month, do you think that other people would have thought of you as a “perfectionist”? |  |  |  |  |
| 11 | Over the past month, have you kept trying to meet your standards, even if this has meant that you have missed out on things? |  |  |  |  |
| 12 | Over the past month, have you avoided any tests of your performance (at meeting your goals) in case you failed? |  |  |  |  |

THANK YOU
